# Adaptability and selectivity of human peroxisome proliferator-activated receptor (PPAR) pan agonists revealed from crystal structures

**DOI:** 10.1107/S0907444909015935

**Published:** 2009-07-10

**Authors:** Takuji Oyama, Kenji Toyota, Tsuyoshi Waku, Yuko Hirakawa, Naoko Nagasawa, Jun-ichi Kasuga, Yuichi Hashimoto, Hiroyuki Miyachi, Kosuke Morikawa

**Affiliations:** a The Takara Bio Endowed Division, Department of Biomolecular Recognition, Institute for Protein Research, Osaka University, Open Laboratories of Advanced Bioscience and Biotechnology, 6-2-3 Furuedai, Suita, Osaka 565-0874, Japan; b Institute of Molecular and Cellular Biosciences, The University of Tokyo, Yayoi, Bunkyo-ku, Tokyo 113-0032, Japan

**Keywords:** peroxisome proliferator-activated receptors, ligand-binding domains, agonists

## Abstract

Peroxisome proliferator-activated receptors (PPARs) belong to the nuclear hormone receptor family, which is defined as transcriptional factors that are activated by the binding of ligands to their ligand-binding domains (LBDs). Although the three PPAR subtypes display different tissue distribution patterns and distinct pharmacological profiles, they all are essentially related to fatty-acid and glucose metabolism. Since the PPARs share similar three-dimensional structures within the LBDs, synthetic ligands which simultaneously activate two or all of the PPARs could be potent candidates in terms of drugs for the treatment of abnormal metabolic homeostasis. The structures of several PPAR LBDs were determined in complex with synthetic ligands, derivatives of 3-(4-alkoxy­phenyl)propanoic acid, which exhibit unique agonistic activities. The PPARα and PPARγ LBDs were complexed with the same pan agonist, TIPP-703, which activates all three PPARs and their crystal structures were determined. The two LBD–ligand complex structures revealed how the pan agonist is adapted to the similar, but significantly different, ligand-binding pockets of the PPARs. The structures of the PPARδ LBD in complex with an α/δ-selective ligand, TIPP-401, and with a related δ-­specific ligand, TIPP-204, were also determined. The comparison between the two PPARδ complexes revealed how each ligand exhibits either a ‘dual selective’ or ‘single specific’ binding mode.

## Abstract 2

The structures of the ligand-binding domains (LBDs) of human peroxisome proliferator-activated receptors (PPARα, PPARγ and PPARδ) in complexes with a pan agonist, an α/δ dual agonist and a PPARδ-specific agonist were determined. The results explain how each ligand is recognized by the PPAR LBDs at an atomic level.

## 1. Introduction

The nuclear hormone receptor family of ligand-activated transcription factors comprises 48 members in humans (Chawta *et al.*, 2001 ▶). The members of one subgroup, the peroxisome proliferator-activated receptors (PPARα, PPARγ and PPARβ/δ), are the key transcriptional regulators in fatty-acid and glucose metabolism (Rosen & Speigelman, 2001 ▶; Lee *et al.*, 2003 ▶). PPARs are activated by metabolites, such as fatty acids, and synthetic ligands and they regulate gene expression by binding to specific DNA response elements located in the enhancer regions of target genes (Banner *et al.*, 1993 ▶; Li *et al.*, 2003 ▶). Each PPAR subtype displays a distinct tissue distribution (Willson *et al.*, 2000 ▶). PPARα is expressed in tissues involved in lipid oxidation, such as liver, heart, muscle and kidney, and it regulates the genes associated with fatty-acid uptake and metabolism. PPARδ, which is expressed ubiquitously, is associated with improved insulin sensitivity and elevated HDL levels. PPARγ is expressed in adipose tissues, macrophages and vascular smooth muscles and directs the regulation of genes related to adipogenesis and lipid storage. PPARs form heterodimers with the retinoid X receptor (RXR) and ligand-bound PPARs adopt an activated conformation (Walczak & Tontonoz, 2002 ▶). Additional co-activator proteins are recruited to create a complex, which coordinates and regulates the expression of large gene arrays (Yu & Reddy, 2007 ▶). Dysfunctional regulation of the expression of these genes leads to a range of human diseases, including atherosclerosis, cancer, diabetes and obesity (Lehrke & Lazer, 2005 ▶). Therefore, PPARs have attracted strong interest from the pharmaceutical industry.

Since the first determination of the crystal structure of the PPARγ LBD, many atomic structures of PPAR LBDs com­plexed with various ligands have been reported (Nolte *et al.*, 1998 ▶; Cronet *et al.*, 2001 ▶; Xu *et al.*, 2002 ▶). Since PPAR activation is substantially dependent on ligand binding, most structural studies have targeted the LBDs. Indeed, synthetic ligands have made great con­tributions to medicine. For example, PPARα agonists such as fenofibrate are clinically used for the treatment of dyslipidaemia, while PPARγ agonists such as pioglitazone are employed for the treatment of type 2 diabetes mellitus. Pharmacological approaches generated the concept of ‘full’ and ‘partial’ agonists, in which the agonists are grouped into classes depending on their transcriptional activities. Several crystal structures support such an idea: the full agonists stabilize the C-terminal helix H12 in the active conformation by directly interacting with a key tyrosine residue on the helix, while the partial agonists interact with amino-acid residues on regions other than helix H12 (Gampe *et al.*, 2000 ▶; Xu *et al.*, 2001 ▶, 2002 ▶; Bruning *et al.*, 2007 ▶). Structural studies have also been oriented toward larger components and complexes with co­activator and co­repressor peptides or with the RXR LBD have been reported. More recently, the intact full-length structure of the PPARγ–RXRα hetero­dimer was reported and pro­vided clearer insights into the activation mechanism of the receptor (Chandra *et al.*, 2008 ▶).

In contrast to synthetic ligands, structural analyses with endogenous ligands or metabolites, including prostaglandin, have been hampered, probably because of the insoluble nature of hydrophobic fatty acids. However, a few groups have surpassed this technical barrier and determined the structures of the LBDs complexed with several lipid metabolites, which have provided im­portant insights into PPARγ activation caused by naturally occurring ligands (Itoh *et al.*, 2008 ▶; Waku *et al.*, 2009 ▶). These studies revealed the interesting finding that receptor activation requires covalent-bond formation between a cysteine residue and the fatty acid and thus led to the proposal of an activation mechanism that is distinct from that by synthetic ligands.

Although many studies have revealed the tissue-specific distributions and distinct roles of the three PPAR subtypes, these three subtypes are essentially related to gene-expression regulation of proteins that are involved in fatty-acid metabolism (Tenenbaum *et al.*, 2005 ▶). If a single compound acts on different PPARs simultaneously, it could exhibit novel pharmacological effects and hopefully modified effects on patients with symptoms of metabolic syndrome. This idea could be possible even in terms of receptor conformations because the LBDs of the three PPARs share significant structural similarity to each other; indeed, it has been reported that some agonists can activate all three PPARs or two of the three subtypes (Tenenbaum *et al.*, 2005 ▶). For example, bezafibrate is an old and well known PPAR pan (α, γ, δ) activator that was the first to be clinically tested. Recently, Artis *et al.* (2009 ▶) developed a novel PPAR pan agonist, indeglitazar, using a combination of biochemical and structural approaches.

**Figure 1:**
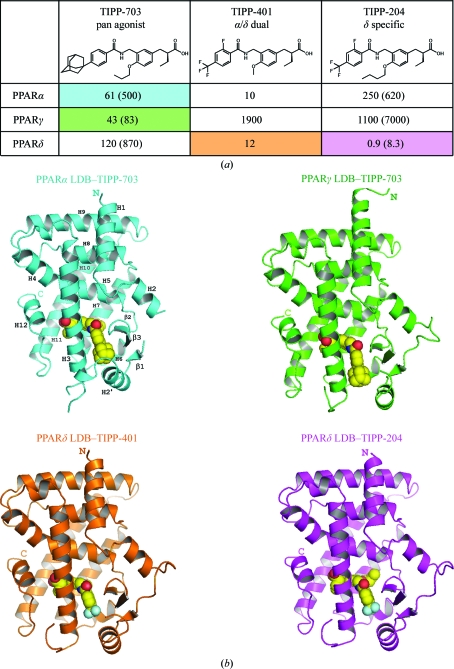
Crystal structures of PPAR LBD–TIPP complexes. (*a*) Chemical formulae of TIPP-703, TIPP-401 and TIPP-204. The numbers indicate the EC_50_ (n*M*), the molar concentration of the compounds that affords 50% of the maximal reporter activity, in our PPAR-GAL4 chimeric reporter assays using transiently transfected HEK-293 cells (Kasuga *et al.*, 2006 ▶). Values in parentheses indicate the activities of the antipodal (*R*) isomers of TIPP-703 and TIPP-401. For the structures of the four complexes determined in this study, the columns in the table are coloured cyan (PPARα LBD–TIPP-703), green (PPARγ LBD–TIPP-703), orange (PPARδ LBD–TIPP-401) and magenta (PPARδ LBD–TIPP-204). This colouring is used throughout the manuscript. (*b*) Overall structures of the complexes. Proteins are represented as ribbon models and the ligands are depicted as space-filling models, with F, C, N and O atoms in aqua, yellow, blue and red, respectively.

Based on our long-standing structure–activity relation (SAR) studies of nuclear receptors, we also succeeded in the synthesis of agonist ligands, TIPP compounds, that have unique functional characteristics with the PPARs, using a series of 3-­(4-alkoxyphenyl)propanoic acid derivatives as lead com­pounds (Miyachi & Hashimoto, 2008 ▶). For example, TIPP-204 specifically activates PPARδ (Kasuga *et al.*, 2007 ▶; Kasuga, Oyama, Nakagome *et al.*, 2008 ▶) and TIPP0401 activates both PPARα and PPARδ (Kasuga *et al.*, 2006 ▶), whereas TIPP-703 can activate all three PPARs (Kasuga, Yamasaki *et al.*, 2008 ▶; Fig. 1 ▶ *a*). The high-resolution crystal structures of the PPARs complexed with these ligands will not only provide the structural basis for the specific or versatile binding mode of these ligands, but also for the further development of drugs that could be candidates for the treatment of altered metabolic homeo­stasis. Here, we describe the crystal structures of human PPAR LBD–TIPP ligand complexes determined at 2.0–3.0 Å resolution. We obtained four of the six possible LBD–ligand complexes between the three PPARs and the three ligands. The PPARα and PPARγ LBDs were both solved in complexes with a pan agonist, TIPP-703. Although the structure of the PPARδ LBD–TIPP-703 complex was not determined in this study, we found a unique binding mode of TIPP-703 to the two PPAR LBDs. TIPP-401 and TIPP-204 possess almost the same structure, except for minor parts. Nonetheless, TIPP-204 can activate both PPARα and PPARδ, while TIPP-401 activates only PPARδ. A detailed structural comparison of the two complex structures bound to PPARδ revealed the different binding modes between the PPARα/δ dual and PPARδ-specific ligands.

## 2. Materials and methods

### 2.1. Protein expression and purification

The recombinant ligand-binding domains (LBDs) of human PPARα (residues 200–468), PPARγ (residues 195–476) and PPARδ (residues 206–477) were expressed as N-terminal His-tagged proteins using the pET28a vector (Novagen). Each expression plasmid was transformed into Rosetta(DE3) pLysS competent cells (Novagen) and the cells were grown in Terrific broth containing 100 µg ml^−1^ kanamycin at 310 K to an OD_600_ of 0.6–0.8. Protein expression was induced with 1 m*M* iso­propyl β-d-1-thiogalactopyranoside for 48 h at 289 K and the cells were then harvested and frozen at 193 K until use.

For the purification of the PPARα LBD, the cells from a 1 l culture were resuspended and disrupted in 25 ml buffer *A* [20 m*M* Tris–HCl pH 8.0, 150 m*M* NaCl, 1 m*M* TCEP, 10%(*v*/*v*) glycerol and a Complete protease-inhibitor cocktail tablet (Roche)]. The soluble fraction was collected by centrifugation at 16 000 rev min^−1^ and 277 K for 20 min. Polyethyleneimine was added to the supernatant to a final concentration of 0.15%(*v*/*v*) to remove nucleic acids derived from the host cells. The soluble fraction was precipitated using 80% saturated ammonium sulfate. The protein was resuspended in the above sonication buffer and loaded onto a HisTrap HP  nickel-chelate column (GE Healthcare); the column was developed with a linear gradient of 0–0.5 *M* imidazole. The pooled fraction was dialyzed against buffer *B* (20 m*M* Tris–HCl, 1 m*M* TCEP and 10% glycerol pH 8.0). Before dialysis, 25 units of thrombin protease were added to cleave the N-­terminal His tag. The solution was loaded onto a HiTrap Q anion-exchange column (GE Healthcare), which was developed with a linear gradient of 0–1 *M* NaCl. The pooled fraction was concentrated to about 5–10 ml and applied onto a HiLoad 26/60 Superdex 75 gel-filtration column (GE Healthcare) equilibrated with buffer *C* (20 m*M* Tris–HCl, 150 m*M* NaCl, 1 m*M* TCEP and 10% glycerol pH 8.0).

In the case of the PPARδ LBD, the cell-lysis supernatant was treated and partially purified as for the PPARα LBD and the protein was further purified by two steps of column chromatography. After the addition of thrombin protease (25 units) to cleave the N-terminal His tag, the pooled fraction from the nickel-chelate column was dialyzed against buffer *D* (20 m*M* MES, 10 m*M* DTT, 100 m*M* ammonium acetate pH 6.0) and loaded onto a HiTrap SP cation-exchange column (GE Healthcare). The protein was eluted with a linear gradient of 0.01–1.0 *M* ammonium acetate. Finally, the protein was purified by gel-filtration chromatography on a HiLoad 26/60 Superdex 75 column eluted with buffer *E* (20 m*M* HEPES, 10 m*M* DTT, 500 m*M* ammonium acetate pH 7.5). The PPARγ LBD was purified as described previously (Waku *et al.*, 2009 ▶).

### 2.2. Crystallization, data collection and model refinement

Crystals of the PPARα LBD–TIPP-703 pan agonist com­plex were obtained by cocrystallization. Firstly, 20 µl 20 m*M* TIPP-703 in 100% DMSO was added to 2.5 ml PPARα LBD solution (approximately 1.2 mg ml ^−1^, 45 µ*M*) and the mixture was incubated for 2 h at 277 K. The complex was concentrated to 7 mg ml^−1^ using a 5000 molecular-weight cutoff Amicon Ultra 4 centrifugal concentrator (Millipore) and was then crystallized using hanging-drop vapour diffusion at 293 K; 1 µl of the above complex solution was mixed with an equal volume of crystallization buffer consisting of 0.1 *M* HEPES pH 7.5 and 25%(*w*/*v*) PEG 3350. Needle-shaped crystals were obtained in a few days.

The two PPARδ LBD–ligand complexes were also obtained by cocrystallization. The complexes were formed by diluting protein solutions (0.1 mg ml^−1^) containing higher concentrations (about three times) of the ligand and the solutions were concentrated to 6–7 mg ml^−1^ prior to crystallization. The PPARδ LBD–ligand complexes were crystallized by hanging-drop vapour diffusion at 293 K with a reservoir solution con­taining 11–14%(*w*/*v*) PEG 4000, 200 m*M* KCl, 40 m*M* bis-tris methane, 6%(*v*/*v*) 1,3-propanediol, 0.5%(*w*/*v*) *n*-heptyl-β-d-glucopyranoside, 1 m*M* EDTA and 1 m*M* CaCl_2_. Diffraction-quality crystals which had a maximum dimension of 50 µm grew within a few days from crystallization drops in which 2 µl of the protein–ligand complex solution was mixed with 1 µl crystallization buffer.

We have previously succeeded in determining the crystal structures of several PPARγ–ligand complexes with endogenous fatty acids and related compounds (Waku *et al.*, 2009 ▶). This study essentially involves the same strategy. We first crystallized the ligand-free PPARγ LBD by hanging-drop vapour diffusion at 293 K using a reservoir solution containing 0.1 *M* HEPES pH 7.5 and 0.8 *M* sodium citrate. The TIPP-703 agonist, at a concentration of 0.1 m*M* in reservoir solution containing 1% DMSO, was then soaked into the unliganded crystals for three weeks.

All crystals were flash-cooled in a liquid-nitrogen stream after briefly soaking them in a cryoprotection buffer  suitable for the PPAR LBD–ligand complex. The crystallization reservoir solution supplemented with 25%(*v*/*v*) glycerol was used for cryoprotection of the PPARα LBD–ligand complex crystals and that supplemented with 20% PEG 1000 was used for the crystals of the PPARδ LBD complexes. In the case of the PPARγ LBD complex crystals, an increase in the sodium citrate concentration from 0.8 to 1.4 *M* and the further addition of 30%(*v*/*v*) glycerol were required for stable cryoprotection. Diffraction data were collected on BL38B1 at SPring-8 (Harima, Japan) and were processed using *HKL*-2000 (Otwinowski & Minor, 1997 ▶). All structures were solved by the molecular-replacement method with the program *CNS* (Brünger *et al.*, 1998 ▶) using the previously published structures as probes. The correctly positioned molecules were refined with *CNS* and *O* (Jones *et al.*, 1991 ▶). Initial atomic models of the TIPP compounds were built using *MOE* (Ryoka Systems Inc.) and topology and parameter files for the refinement were generated by the *HIC-Up* server (Kleywegt, 2007 ▶). The crystallographic data and refinement statistics are summarized in Table 1 ▶.

## 3. Results and discussion

### 3.1. TIPP agonist ligands used in this study

The three TIPP agonists used in this study were developed based on our SAR results. They share a common formula, with a head part containing the carboxyl group, a central and a tail benzene ring, with a linking group between the two benzene rings (Fig. 1 ▶ *a*). TIPP-703 (pan agonist), (*S*)-2-{3-[(4-adamantan-1-ylbenzoylamino)methyl]-4-propoxybenzyl}butyric acid, has a prominent adamantyl group at the *para* position of the tail benzene ring and a propoxy group on the central benzene ring. TIPP-401 (α/δ dual agonist), (*S*)-2-{3-[(2-­fluoro-4-trifluoro­methylbenzoylamino)methyl]-4-methoxy­benzyl}butyric acid, and TIPP-204 (δ-specific agonist), (*S*)-2-{4-butoxy-3-[(2-fluoro-4-trifluoromethylbenzoylamino)methyl]benzyl}butyric acid, have a trifluoromethyl (CF_3_) group at the *para* position of the tail benzene instead of the adamantane in TIPP-703 and an additional fluorine at the *ortho* position. The only difference between TIPP-401 and TIPP-204 is the length of the carbon chain on the central benzene ring. TIPP-401 has a methyl group, while TIPP-204 has an *n*-butoxy chain. Considering the TIPP ligand-activation ability, there are six possible complexes between the three PPAR subtypes and the three TIPP ligands. We tried to crystallize all of the PPAR LBD complex crystals and consequently obtained and determined four types of complex crystals. Based on a detailed comparison of these four complexes, we were able to ascertain the adaptability and selectivity of each TIPP ligand.

### 3.2. Crystallization and structure determination

We performed high-throughput crystallization screening using a Mosquito automated nanodrop dispenser (TTP Labtech). Typically, a 200 nl aliquot of the protein–ligand complex solution was mixed with an equal volume of reservoir solution from commercially available screening kits. We generated duplicate plates for each screening kit and placed one in a room-temperature (293 K) incubator and the other in a cold (277 K) incubator.

Crystals of the PPARα LBD–TIPP-703 complex that diffracted to high resolution were obtained using this screening strategy. The complexes crystallized under more than ten conditions of the 1728 in the semi-automatic setup, indicating that this complex has sufficient structural stability to be subjected to structure analysis (data not shown). Under these conditions, we reproducibly obtained diffraction-quality crystals using the hanging-drop vapour-diffusion method in the traditional manual setup, in which 1 µl complex solution was mixed with an equal volume of reservoir solution and equilibrated against 500 µl reservoir solution. Although several crystal structures of PPARα–ligand complexes have been published, the present PPARα–TIPP-703 complex was crystallized as a novel crystal form. In contrast, the present screening did not allow us to produce any novel crystal forms of the PPARδ LBD–ligand and PPARγ LBD–ligand com­plexes that were suitable for structure analysis. They were successfully crystallized under the previously reported conditions and therefore straightforward structure determinations were possible (Waku *et al.*, 2009 ▶; Fytte *et al.*, 2006 ▶).

The three crystals of the PPARα and PPARδ LBD–ligand complexes were obtained by cocrystallization and the bound ligands within the binding pockets were clearly observed in the electron-density maps. Thus, we could efficiently build the atomic models of the ligands and achieved rapid convergence. On the other hand, as for the PPARγ LBD–TIPP-703 com­plex, we had to collect several sets of X-ray diffraction data in order to determine the structure of the bound pan agonist unambiguously. To obtain the high-quality crystal structure of the PPARγ LBD–TIPP-703 complex, a three-week soaking of the ligand into the crystal was required. Even in the best structures determined at 2.4 Å resolution, the propoxy group located at the centre of TIPP-703 in the PPARγ LBD complex was disordered. Nonetheless, the quality of the complex structure is sufficient to discuss the adaptability of this pan agonist to the three PPARs.

### 3.3. Overview of the PPAR LDB–agonist ligand complexes and comparisons with other PPAR LBD–ligand complexes

All of the PPAR LBDs fold into a three-layered sandwich comprising mainly α-helices, as also observed in the nuclear receptor LBDs. The PPARα LBD–TIPP-703 crystal contained one complex in the asymmetric unit, while the crystals of  PPARγ LBD–TIPP-703, PPARδ LBD–TIPP-401 and PPARδ LBD–TIPP204 contained two molecules. In the two PPARδ LBD structures, the protein–ligand interactions were essentially the same in the two protomers in the asymmetric unit. On the other hand, the two protomers in the asymmetric unit of the PPARγ LBD–TIPP-703 complex exhibited slightly different conformations at the C-terminus, leading to the loss of several protein–ligand contacts (Waku *et al.*, 2009 ▶). How­ever, the overall interaction modes of the proteins with the ligands were substantially conserved.

The ligand-binding sites of the PPAR LBDs generally exhibit Y-shaped pockets. The deepest arm is located behind helix 3, where the bound ligands contact both hydrophilic and hydrophobic residues. In particular, the hydrophilic patch contains the Tyr residue on the AF-2 helix (helix 12), which plays a key role in interacting with the carboxyl group of the ligands. The conformation of the AF-2 helix generally plays a crucial role in the regulation of coactivator segment binding (Nolte *et al.*, 1998 ▶). The mostly hydrophilic second arm is located on the opposite side to the first arm, against helix 3. The hydrophobic third arm also lies at the entrance to the binding pocket.

**Figure 2:**
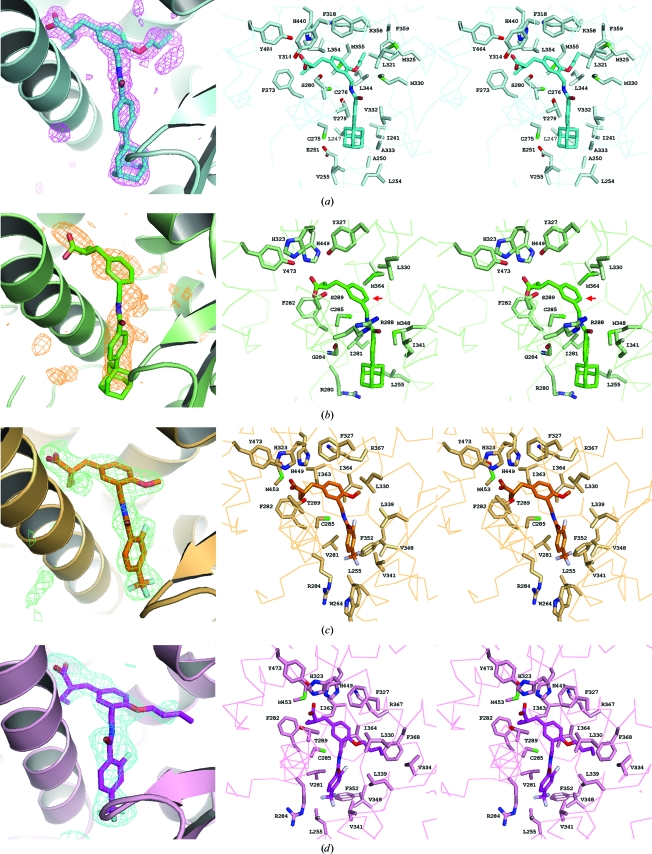
Close-up views of the ligand-binding pockets. (*a*) PPARα LBD–TIPP-703. (*b*) PPARγ LBD–TIPP-703. (*c*) PPARδ LBD–TIPP-401. (*d*) PPARδ LBD–TIPP-204. The left column shows the OMIT *F*_o_ − *F*_c_ electron-density maps (contoured at 2.2σ) and the right columns show stereoviews of the interaction between the ligand-binding pockets and the bound ligands. The amino-acid residues contacting the ligands are labelled.

The present four complexes generally show very similar TIPP ligand-binding modes. The head carboxyl groups are located in the first cavity, making conventional interactions with the polar side chains, and thus all of the PPAR LBDs adopt the conventional active conformation of helix 12 (Fig. 2 ▶). The central benzene rings and the alkoxy groups are located in the centre and the second cavity, respectively, and the tail benzene and the additional groups lie at the entrance.

A database examination revealed that our PPAR LBD–TIPP complex structures resemble those of PPARα with AZ 242 (PDB code 1i7g; Cronet *et al.*, 2001 ▶) and with a PPARα/γ dual agonist, α-acyl-β-phenylpropanioic acid (PDB code 2npa; Han *et al.*, 2007 ▶), of PPARγ with rosiglitazone (PDB code 2prg; Nolte *et al.*, 1998 ▶) and with a PPARα/γ dual agonist, a phenylpropanoic acid derivative (PDB code 2q8s; Casimiro-Garcia *et al.*, 2008 ▶), and of PPARδ with GW2331 (PDB code 1y0s; Takada *et al.*, 2000 ▶) and with a 3,4,5-trisubstituted isoxazole (PDB code 2j14; Epple *et al.*, 2006 ▶) (see Supplementary Fig. 2^1^). Although the detailed binding modes of the PPAR ligands differ depending on their chemical and pharmacological characteristics, the carboxyl group head of the ligands usually occupies a common position with similar conformations. In fact, the local similarity of the ligand-binding mode is even apparent between our TIPP ligands and natural fatty acids (Fytte *et al.*, 2006 ▶; Itoh *et al.*, 2008 ▶; Waku *et al.*, 2009 ▶).

### 3.4. Adaptability of the TIPP-703 pan agonist

In our previous SAR study, the introduction of an adamantyl residue at the *para* position of the tail benzene ring drastically improved the pan-agonism of the com­pounds, particularly for PPARγ (Kasuga, Yamasaki *et al.*, 2008 ▶). Several adamantane-containing compounds exhibited EC_50_ values, as estimated by our cell-based assay, in the submicromolar range or lower. In contrast, when the adamantyl moiety was replaced by a trifluoromethyl group, the EC_50_ values against PPARγ increased to a micromolar or higher value. The present structural analyses highlight the effect of the adamantyl residue on the pan-agonism.

**Figure 3:**
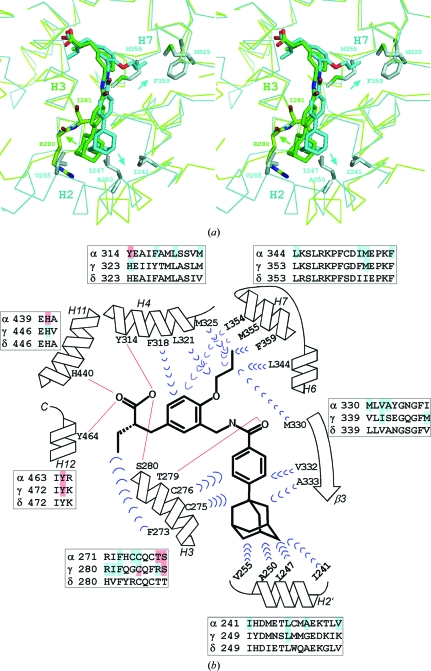
Comparison between the PPARα LBD–TIPP-703 and the PPARγ LBD–TIPP-703 complexes. (*a*) Stereoview of the superimposed structures. The PPARα LBD–TIPP-703 complex is coloured cyan and the PPARγ LBD–TIPP-703 complex is coloured green. The key contact residues in the complexes are highlighted. Prominent interactions in each complex are indicated by arrows. (*b*) Schematic view of the protein–ligand interactions. A structure-based sequence alignment was generated around the ligand-binding pocket. In the PPARα and PPARγ sequences, the residues that interact with TIPP-703 are coloured blue (hydrophobic) and red (hydrophilic).

A close-up view of the superimposed structures of the PPARα and PPARγ LBDs complexed with TIPP-703 is shown in Fig. 3 ▶(*a*). The overall binding modes of the pan agonist are similar between the two PPARs, but significant differences are observed in the protein–ligand interaction. In the PPARα LBD–TIPP-703 complex, the adamantyl group mainly contacts hydrophobic residues from the H2′ helix (Ile241, Leu247, Ala250 and Val255) and from the β3 strand (Val332 and Ala333) at the entrance of the ligand-binding pocket. In contrast, in the PPARγ LBD complex the same group mainly interacts with Arg280 and Ile281 on the H3 helix. Furthermore, in the PPARα LBD complex the propoxy group of TIPP-703 in the second cavity of the ligand-binding pocket is well ordered in the crystal and interacts with the three hydrophobic residues Met325, Met355 and Phe359. In the PPARγ LBD complex, the propoxy group of TIPP-703 should occupy a similar position to that in the PPARγ LBD complex. However, we did not observe any significant electron density corresponding to the propoxy group of TIPP-703, although the ligand molecule was mostly ordered in the crystal with an average *B* factor of 65.8 Å^2^. This indicates that the propoxy group interacts weakly with PPARγ in the ligand-binding pocket. Interestingly, the observed binding features are highly consistent with our previous analyses: the length of the alkoxy group (methoxy, ethoxy and propoxy) on the central benzene ring did not drastically affect the affinity towards PPARγ, indicating the lower contribution of the alkoxy group to the affinity of TIPP-703 towards PPARs.

The PPARδ LBD exhibits 80 and 65% identity to those of PPARα and PPARγ, respectively, indicating that PPARδ is slightly more similar to PPARα than to PPARγ (Fig. 3 ▶ *b* and Supplementary Fig. 1 ▶ 1). Accordingly, TIPP-703 could bind to PPARα and PPARγ in a similar manner.

### 3.5. Selectivity between the α/δ dual agonist and the δ-­specific agonist

In contrast to TIPP-703, the dual selectivity of TIPP-401 and the PPARδ-specific affinity of TIPP-204 are attributed to the chain lengths of the alkoxy groups at the centre of the ligand molecules. In the structure of the PPARδ LBD–TIPP401 α/δ dual agonist complex, the methoxy group is oriented toward the second small cavity but makes fewer interactions with the surrounding amino-acid residues (Fig. 2 ▶ *c*). When the methoxy group is replaced by an *n*-butoxy chain, TIPP-401 changes to the δ-specific compound TIPP-204, in which the extended alkoxy group forms a hydrophobic interaction with the Val334 side chain using the distal methyl group (Fig. 2 ▶ *d*). This interaction indeed improved the activation of PPARδ (Fig. 1 ▶ *a*).

**Figure 4:**
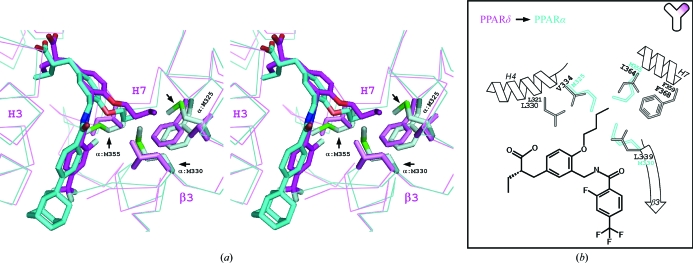
Comparison between the PPARα LBD–TIPP-703 and the PPARδ LBD–TIPP-204 complexes. (*a)* Stereoview of the superimposed structures. The PPARα LBD–TIPP-703 complex is coloured cyan and the PPARδ LBD–TIPP-204 complex is coloured magenta. The key contact residues of the complexes are highlighted. Prominent interactions in each complex are indicated by arrows. (*b*) Schematic view of the protein–ligand interactions, highlighting the specificity of TIPP-204 toward PPARδ.

On the other hand, the situation is opposite in PPARα. In the second binding cavity, the three amino acids of PPARδ, Val334, Leu339 and Ile364, are replaced by larger amino acids, Met325, Met330 and Met355, and thus a smaller second binding cavity exists in PPARα than in PPARδ (Fig. 4 ▶). Therefore, the methoxy group of TIPP-401 is suitable for interaction with PPARα. When the methoxy group is replaced by the longer *n*-butoxy group, TIPP-401 becomes TIPP-204 and PPARα Met325 and Met330 should be too close and cause a steric clash with the *n*-butoxy group, even though some conformational change could occur in both the PPARα Met325 side chain and the *n*-butoxy group of TIPP-204 (Fig. 4 ▶). Considering the results of our previous SAR study and the current structural analyses, the distal single C atom could be unfavourable for PPARα (Kasuga *et al.*, 2007 ▶; Fig. 1 ▶ *a*). The EC_50_ value of the transactivation activity of TIPP-401 against PPARα was 10 n*M* and that of another compound with an *n*-­propoxy group at the same position was 41 n*M*, still indicating strong activity. This is also supported by the PPARα LBD–TIPP-703 pan agonist complex structure. TIPP-703 has an *n*-propoxy group at the corresponding site and this group lies in the second binding cavity with favourable contacts to PPARα (Fig. 2 ▶ *a*). In contrast, the EC_50_ value of TIPP-204 with an *n*-butoxy group for PPARα was 250 n*M*, which is about 25 times higher than that of TIPP-401.

Our previous mutational analysis also supports the present findings (Kasuga, Oyama, Nakagome *et al.*, 2008 ▶). When the three key residues (Val334, Leu339 and Ile364) of PPARδ that form the interaction with TIPP-204 were replaced by Met residues, the transactivation activities of the PPARδ mutants by TIPP-204 were decreased. Conversely, when the three methionines of PPARα corresponding to the above hydrophobic residues were replaced by other residues (M325V, M330L and M355I; Fig. 4 ▶), the activities of the mutants induced by TIPP-204 were improved.

### 3.6. Development of the TIPP compounds from a PPARα-specific ligand

The TIPP-401 α/δ dual agonist was developed from a PPARα-specific agonist, KCL, by changing the linking group between the central and tail benzene rings and by introducing an F atom at the 2-position of the tail benzene ring (Kasuga *et al.*, 2006 ▶). Owing to the introduction of the F atom in particular, TIPP-401 exhibited a more potent transactivation activity against PPARδ than KCL: 170 and 12 n*M*, respectively. As expected, the PPARδ LBD–TIPP-401 complex structure exhibited a prominent interaction between the protein and the ligand *via* the F atom (Fig. 2 ▶ *c*). This F atom makes van der Waals interactions with the geminal dimethyl groups of the side chain of Leu339. The corresponding Met330 of PPARα and Val339 of PPARγ do not interact with the atoms on the tail benzene ring of TIPP-703 without the F atom (Figs. 2 ▶ *a* and 2 ▶ *b*), highlighting the effect of the F atom.

The present TIPP compounds have an ethyl group with an (*S*)-configuration at the α-position of the head carboxyl group and all of the compounds exhibit more potent transactivation activity than their antipodal (*R*) isomers (Kasuga *et al.*, 2007 ▶). Notably, the present structural study revealed the enantio­selectivity of the TIPP compounds. The ethyl groups are located at the deepest binding sites with the head carboxyl groups and contact the surrounding hydrophobic residues. When their *R* isomers approach the ligand-binding pocket, the ethyl groups may cause a steric clash with the surrounding residues, particularly those on the central parts of the H3 helix, even though some structural rearrangements could occur in both the proteins and ligands.

**Figure 5:**
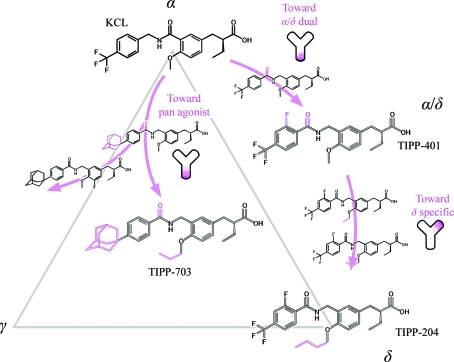
Summary of the development of the present TIPP compounds from a PPARα-specific agonist, KCL. The TIPP agonists used in this study were all developed from KCL using structure–activity relation (SAR) studies. When specific chemical groups, coloured magenta, were introduced into the ligand compounds, the transactivation abilities were drastically changed. The modification effects of the agonist ligands were found to increase the protein–ligand interactions at specific positions in the ligand-binding pockets.

In summary, the present crystal structures revealed our successive logical design of the TIPP compounds from a PPARα-specific ligand, KCL, at the atomic level. The history of the development is summarized in Fig. 5 ▶. It is expected that more effective ligands with unique characteristics could be developed from the current X-ray crystallographic study. For example, another PPAR pan agonist which exhibits more potent transactivation activity than TIPP-703 has been synthesized (Kasuga, Oyama, Hirakawa *et al.*, 2008 ▶).

## Supplementary Material

PDB reference: PPARα LBD–TIPP-703, 2znn, r2znnsf

PDB reference: PPARγ LBD–TIPP-­703, 2zno, r2znosf

PDB reference: PPARδ LBD–TIPP-401, 2znp, r2znpsf

PDB reference: PPARδ LBD–TIPP-­204, 2znq, r2znqsf

Supplementary material file. DOI: 10.1107/S0907444909015935/mh5021sup1.pdf

## Figures and Tables

**Table 1:** Crystallographic data and refinement statistics Values in parentheses are for the last shell.

	PPARα–TIPP-703	PPARγ–TIPP-703	PPARδ–TIPP-401	PPARδ–TIPP-204
	Pan agonist	Pan agonist	α,δ dual	δ-specific
Data collection				
Space group	*P*2_1_	*C*2	*P*2_1_	*P*2_1_
Unit-cell parameters				
*a* (Å)	44.372	93.307	39.492	39.172
*b* (Å)	61.529	61.604	93.149	91.947
*c* (Å)	53.124	118.973	96.370	96.361
β (°)	106.290	103.640	97.480	98.010
Wavelength (Å)	1.00000	1.00000	1.00000	1.00000
Resolution (Å)	35.0–2.00 (2.07–2.00)	50.0–2.40 (2.49–2.40)	50.0–3.00 (3.11–3.00)	50.0–2.65 (2.74–2.65)
No. of unique reflections	18142 (1646)	25166 (2065)	13670 (1178)	19488 (1827)
Completeness (%)	98.7 (90.8)	97.1 (80.3)	97.8 (84.9)	99.0 (93.3)
*I*/σ(*I*)	10.6 (3.3)	15.3 (2.7)	7.7 (2.1)	8.6 (2.7)
Redundancy	3.7 (3.1)	3.5 (2.8)	3.7 (2.9)	3.7 (3.1)
*R*_merge_† (%)	6.4 (22.2)	4.1 (26.1)	8.8 (29.7)	9.3 (28.2)
Refinement				
Resolution range (Å)	35.0–2.00	50.0–2.40	38.0–3.00	42.4–2.65
*R*_work_‡/*R*_free_§	21.4/25.3	24.0/28.6	23.0/28.8	21.4/27.6
No. of atoms				
Protein	2054	4111	4198	4215
Water	146	46	5	83
Ligand	37	66	113	98
Average *B* factor (Å^2^)				
Protein	24.74	49.32	42.46	31.73
Water	29.68	41.22	43.66	30.02
Ligand	28.45	65.85	25.22	31.76
R.m.s.d.				
Bond lengths (Å)	0.008	0.010	0.010	0.008
Angles (°)	1.2	1.3	1.4	1.3
PDB code	2znn	2zno	2znp	2znq

† *R*_merge_ = 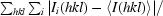 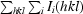, where 〈*I*(*hkl*)〉 is the mean *I*(*hkl*) over symmetry-equivalent reflections.

‡ *R*_work_ = 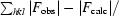 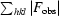, where *F*_obs_ and *F*_calc_ are the observed and calculated structure factors, respectively.

§ *R*_free_ was calculated using 5% of the total reflections, which were chosen randomly and omitted from the refinement.

## Footnotes

1 Supplementary material has been deposited in the IUCr electronic archive (Reference: MH5021). Services for accessing this material are described at the back of the journal.
