# Supplementary material for: Adaptability and selectivity of human peroxisome proliferator-activated receptor (PPAR) pan agonists revealed from crystal structures
Source: Acta Crystallogr D Biol Crystallogr. 2009 Jul 10;65(Pt 8):786–95. doi: 10.1107/S0907444909015935 (PMC2714719; doi:10.1107/S0907444909015935)
Supplement: Supplementary file 1 [file d-65-00786-sup1.pdf]

Supplementary Materials

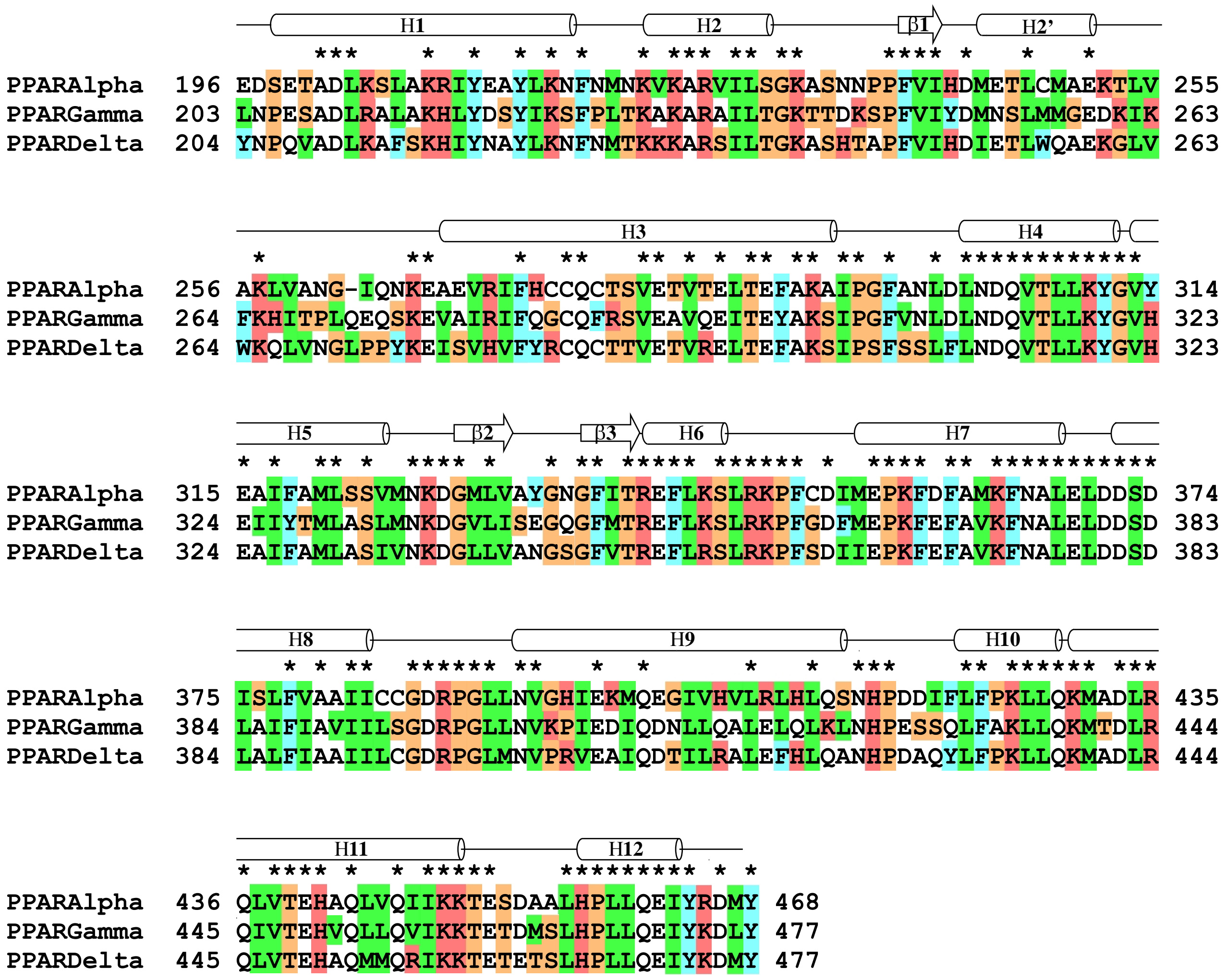

Supplementary Figure 1

Structure-based sequence alignment of the human PPAR ligand binding domains. Amino acid residues are colored according to their characteristics (aromatic, cyan; hydrophobic, green; basic, brown; ambivalent, orange). Secondary structural elements are indicated on the top of the sequences. Conserved residues are highlighted by asterisks.

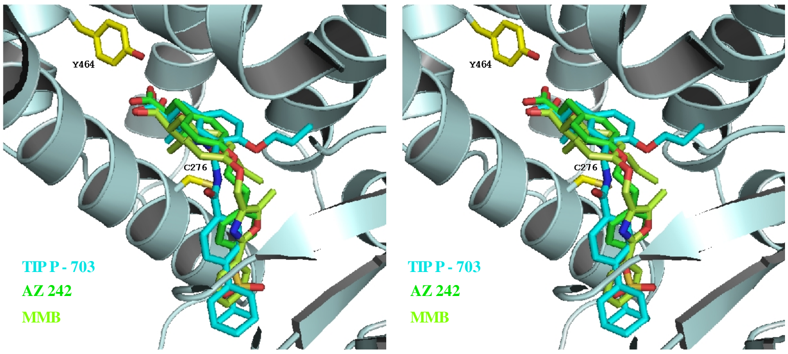

(*a*)

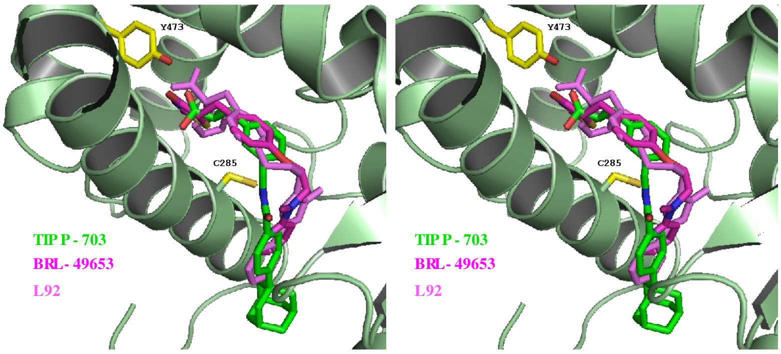

(*b*)

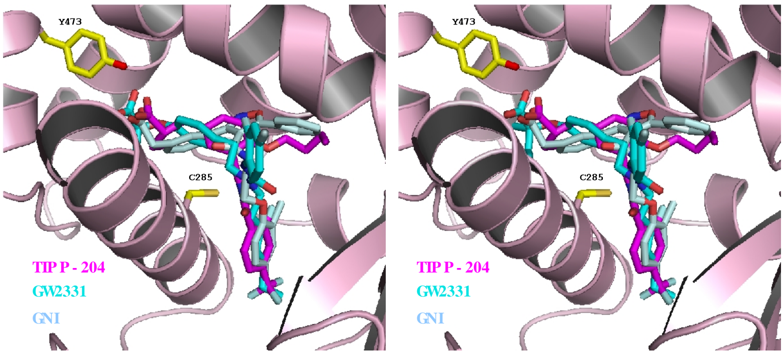

(*c*)

Supplementary Figure 2

Comparison of the PPAR LBD—TIPP ligand complexes with other related structures. (*a*) Superposition of the PPARα LBD cocrystal structures with TIPP-703 (cyan), AZ242 (green), and (2*R*,3*E*)-2-{4-[(5-methyl-2-phenyl-1,3-oxazol-4-yl)methoxy]benzyl}-3-(propoxyimino)butanoic acid (MMB; yellow green). (*b*) Superposition of PPARγ LBD structures in complex with TIPP-703 (green), rosiglitazone (BRL-49653; magenta), and (2*S*)-3-{4-[3-(5-methyl-2-phenyl-1,3-oxazol-4-yl)propyl]phenyl}-2-(1*H*-pyrrol-1-yl)propanoic acid (L92; pink). (*c*) The PPARδ LBD complex structures with TIPP-204 (magenta), GW2331 (cyan), and (3-{4-[2-(2,4-dichloro-phenoxy)-ethylcarbamoyl]-5-phenyl-isoxazol-3-yl}-phenyl)-acetic acid (GNI: light cyan).
